# Intrinsic Functional Plasticity of the Sensory-Motor Network in Patients with Cervical Spondylotic Myelopathy

**DOI:** 10.1038/srep09975

**Published:** 2015-04-21

**Authors:** F. Q. Zhou, Y. M. Tan, L. Wu, Y. Zhuang, L. C. He, H. H. Gong

**Affiliations:** 1 Department of Radiology, the First Affiliated Hospital, Nanchang University, Nanchang, Jiangxi Province, 330006, PRC; 2 Jiangxi Province Medical Imaging Research Institute, Nanchang, Jiangxi Province, 330006, PRC; 3 Department of Oncology, The Second Hospital of Nanchang, Nanchang, Jiangxi Province, 330003, PRC; 4 Department of Orthopaedics and Traumatology, Li Ka Shing Faculty of Medicine, The University of Hong Kong, Pokfulam, Hong Kong

## Abstract

Several neuroimaging studies have suggested brain reorganisation in patients with cervical spondylotic myelopathy (CSM); however, the changes in spontaneous neuronal activity that are associated with connectedness remain largely unknown. In this study, functional connectivity strength (FCS), a data-driven degree centrality method based on a theoretical approach, was applied for the first time to investigate changes in the sensory-motor network (SMN) at the voxel level. Comparatively, CSM not only showed significantly decreased FCS in the operculum-integrated regions, which exhibited reduced resting-state functional connectivity (rsFC) around the Rolandic sulcus, but it also showed increased FCS in the premotor, primary somatosensory, and parietal-integrated areas, which primarily showed an enhanced rsFC pattern. Correlation analysis showed that altered FCS (in the left premotor-ventral/precentral-operculum, right operculum-parietale 4, and right S1) was associated with worsening Japanese Orthopaedic Association scores and that the rsFC pattern was influenced by cervical cord micro-structural damage at the C2 level. Together, these findings suggest that during myelopathy, the intrinsic functional plasticity of the SMN responds to the insufficient sensory and motor experience in CSM patients. This knowledge may improve our understanding of the comprehensive functional defects found in CSM patients and may inspire the development of new therapeutic strategies in the future.

Cervical spondylotic myelopathy (CSM) is one of the most common spinal cord disorders that causes motor and sensory deficits of the limbs^1^ This disease is sometimes regarded as a specific form of incomplete spinal cord injury (SCI)^1^,^2^. In patients with cervical myelopathy, the dysfunction is predominantly attributed to the local injury of the ascending or descending spinal cord fibre^1^,^3^. However, in recent task-functional magnetic resonance imaging (fMRI) studies, cortical reorganisation has been observed to include features such as increased activation in the primary motor and premotor cortices^4^,^5^,^6^ or the loss of activation in the sensory cortex in patients with spinal cord compression^6^. Moreover, several authors reported that central functional adaptations^4^,^6^ or recruitment^4^,^6^,^7^ accompany post-surgical improvements in sensorimotor function in CSM patients. The above evidence demonstrates that functional alterations occur in the sensorimotor cortex and participate in the pathological course of CSM.

The above-mentioned findings provide a basic understanding of the functional response capability of cerebral motor and/or sensory neurons during the performance of experimental manipulations or tasks in CSM patients. The interpretation of these results is limited by spinal cord self-dysfunction and inter-subject variability in task performance, which are the main caveats of this study. Therefore, task-fMRI is not sufficiently objective to evaluate the status of the central nervous system in an individual with a disability such as CSM. Task-fMRI also ignores evaluations of intrinsic functional organization, which involve information processing and integration. Moreover, questions remain concerning the motor and sensory areas in CSM patients. For example, the impact of cervical myelopathy on the intrinsic functional connectivity architecture of sensorimotor networks (SMNs) has not been determined, and whether these alterations in intrinsic activity are harmful or promote functional recovery in CSM patients is not known.

In contrast to task-fMRI, resting-state fMRI (rs-fMRI) requires no stimulation or response, and it can be employed to explore 95% of the energy consumption of intrinsic neuronal activity^8^. Thus, rs-fMRI is an effective platform to explore neuronal functional connectivity across the brain^8^,^9^ and to further understand the pathophysiological mechanisms of intrinsic activity in neurological or psychiatric diseases^8^,^10^. *Functional connectivity strength (FCS)*, a data-driven graph theoretical approach, is measured with degree centrality (DC) of the weighted functional network at the voxel level^11^. In contrast to regional functional analysis (amplitude of low frequency fluctuations^12^ or regional homogeneity^13^) and hypothesis-driven functional connectivity^14^ analysis, this approach provides a strength property by measuring the node's direct connections with a higher spatial resolution^11^,^15^,^16^. In this study, we employed FCS to reveal potential differences in the connectedness of the SMN in a group of CSM patients compared to a group of healthy subject controls (HSCs). In addition, we used seed-based resting-state functional connectivity (rsFC) analysis to reveal the details of the functional networks that are associated with the influenced regions mentioned above (see the flowchart in Figure 1). Together, these results could reveal abnormalities of connectedness across the SMNs of CSM patients in terms of functional plasticity and reorganisation.

## Results

### Demographic and clinical data profiling

Among the 31 patients with CSM, 12 (38.7%) presented with pain or discomfort in the neck or shoulder; 21 (67.7%) presented with weakness, a loss of dexterity, or sensory loss in the upper limbs; 13 (41.9%) had gait instability; 11 (35.5%) had a single-level compression and 20 (64.5%) had two or more levels of compression secondary to disk degeneration, spondylosis, or both. The demographic and clinical data of the study groups are shown in Table 1. There were no significant differences between the groups with respect to age (*P* = 0.764) or sex (*P* = 1.0). However, there were significant differences between the CSM and HSC groups in the Japanese Orthopaedic Association (JOA) scores, Neck Disability Index (NDI) scores and fractional anisotropy (FA) values in the cervical cord. The mentioned clinical measures revealed the disabilities induced by cervical compressive myelopathy (i.e., the loss of the hand dexterity, gait dysfunction), the seriousness of neck-related syndrome and the extent of local damage to the cervical cord. Finally, 29 patients received surgical decompression to relieve their clinical symptoms at the First Affiliated Hospital of Nanchang University, and two patients received surgical procedures at another hospital.

### Functional connectivity strength comparison

We generated voxel-wise FCS within the SMN and further investigated differences between the CSM and HSC groups using rs-fMRI data. The results were highly similar, including the FCS spatial distribution maps and the between-group differences, and they did not depend on the different correlation thresholds considered (*r_0_* = 0.1, 0.15, 0.2, 0.25, 0.3, 0.35, 0.4). The classical reference *r_0_*values^15^,^17^,^18^ are primarily reported in the results that were thresholded using an *r_0_* = 0.25, and the corresponding connection's statistical significance threshold was set at *P* < 0.001 in this study. Other results from voxel-wise FCS using different thresholds are shown in the Figure S1.

**Figure 2:**
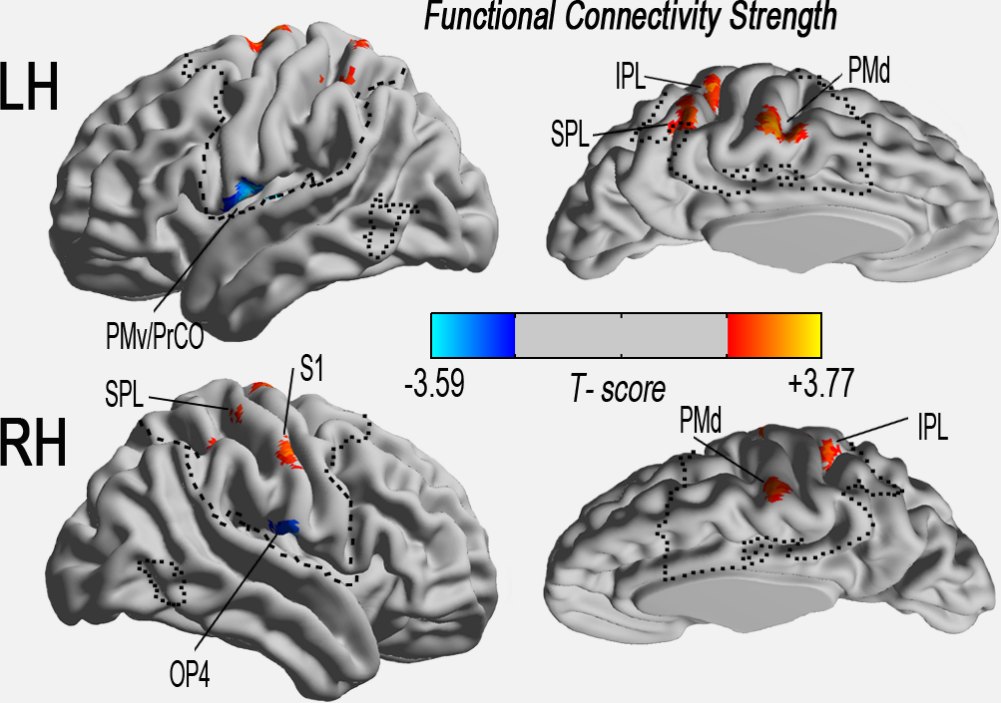
Altered functional connectivity strength (FCS) of the sensory-motor network measured by degree centrality in cervical spondylotic myelopathy (CSM) patients compared to healthy controls. Spatial distribution of significant changes in the CSM (*P* < 0.05, AlphaSim corrected critical cluster size was k = 20), visualised with a surface brain using Brainnet Viewer software (www.nitrc.org/projects/bnv/). The colour bars indicate the t-scores. Yellow-red and cyan-blue colours denote increased and decreased FCS in CSM patients, respectively.*Note: CSM = cervical spondylotic myelopathy; FCS = functional connectivity strength; HSC = healthy subject control; IPL = inferior parietal lobule; k/k_0_ = normalised FCS; LH = left hemisphere; MNI = Montreal Neurological Institute; MTG = middle temporal gyrus; OP4 = operculum parietale 4; PMv = premotor ventral; PrCO = precentral operculum; PMd = premotor dorsal; RH = right hemisphere; RO = rolandic operculum; PreG = precentral gyrus; PostG = postcentral gyrus; S1 = primary somatosensory complex; SPL = superior parietal lobule; SMA = supplementary motor area. The same abbreviations were used for all of the figure and tables.*

Compared with the HSC group, the CSM group showed significantly decreased FCS in the left premotor ventral/precentral operculum (PMv/PrCO) and right operculum parietale 4 (OP4); the CSM group also showed significantly increased FCS in the left and right inferior parietal lobule (IPL), left and right superior parietal lobule (SPL), left and right premotor dorsal (PMd, caudal), and right primary somatosensory complex (S1, BA3A, hand) (Table 2 and Figure 2).

### Clinical metrics associated with the FCS of the SMN

**Figure 3:**
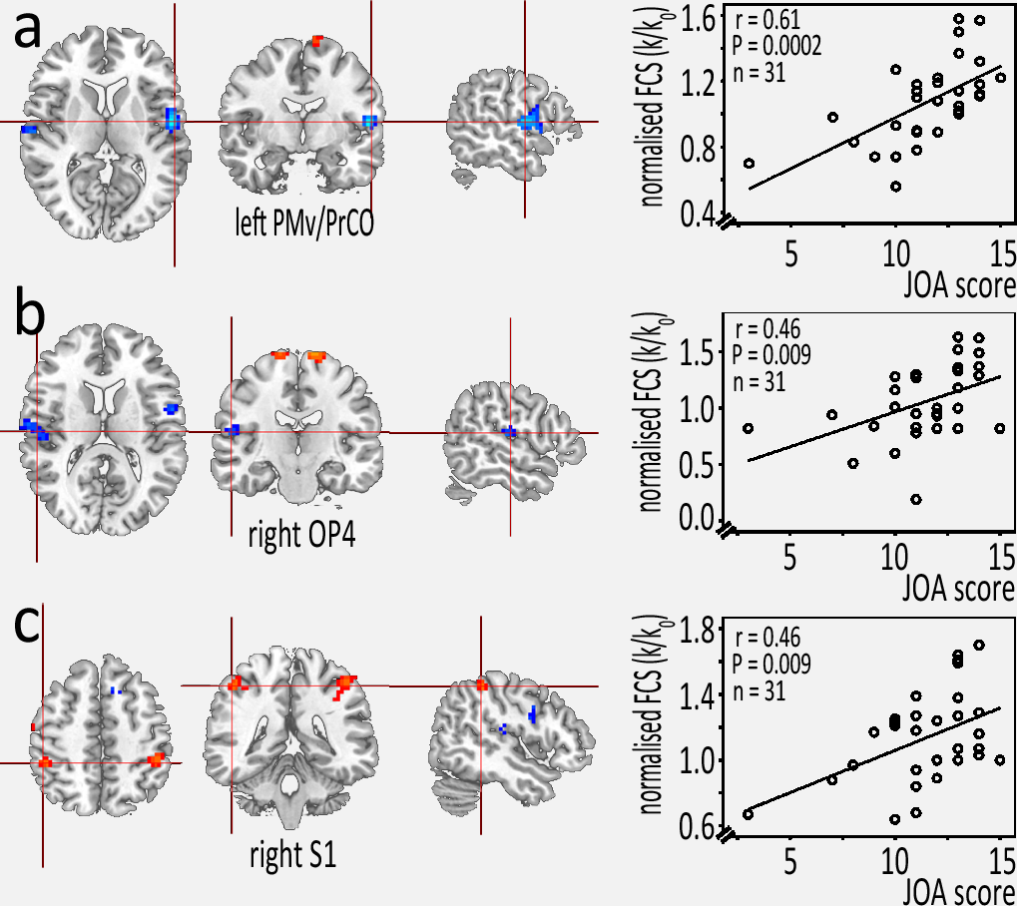
Correlation between the JOA score and the mean-normalized FCS in the left PMv/PrCO, right OP4, and right S1 (representation of the hand) in CSM patients.

In the CSM group, the mean normalized-FCS in the left PMv/PrCO, right OP4, and right S1 showed a significantly positive correlation with the JOA score (*P* < 0.01; Figure 3). Additionally, the mean normalized-FCS in the left IPL (*r* = −0.346; *P* = 0.056) and right S1 (*r* = 0.325; *P* = 0.074) showed a trend or moderate correlation with the FA values at the C2 level. In contrast, there was no significant relationship between the mean normalized-FCS values and other clinical metrics such as the NDI score.

### rsFC network comparison and clinical associations

**Figure 4:**
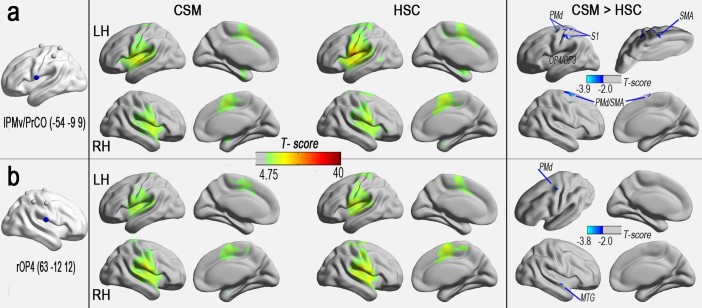
Significant positive rsFC patterns (*P* < 0.001, FDR corrected) of the seed regions with decreased FCS were observed, as were significant differences (*P* < 0.05, AlphaSim corrected critical cluster size was k = 24 in Fig 4a and 4b) between the CSM and HSC groups. *Left column: seed regions with decreased FCS in patients with CSM; middle column: rsFC patterns connected with the seed regions in both the CSM and HSC groups; right column: group differences in rsFC between the two groups*. The colour-bars indicate the t-scores. Yellow-red colours denote highly positive rsFC in one-sample *t*-tests (one group), and cyan-blue colours denote decreased rsFC in CSM patients.

**Figure 5:**
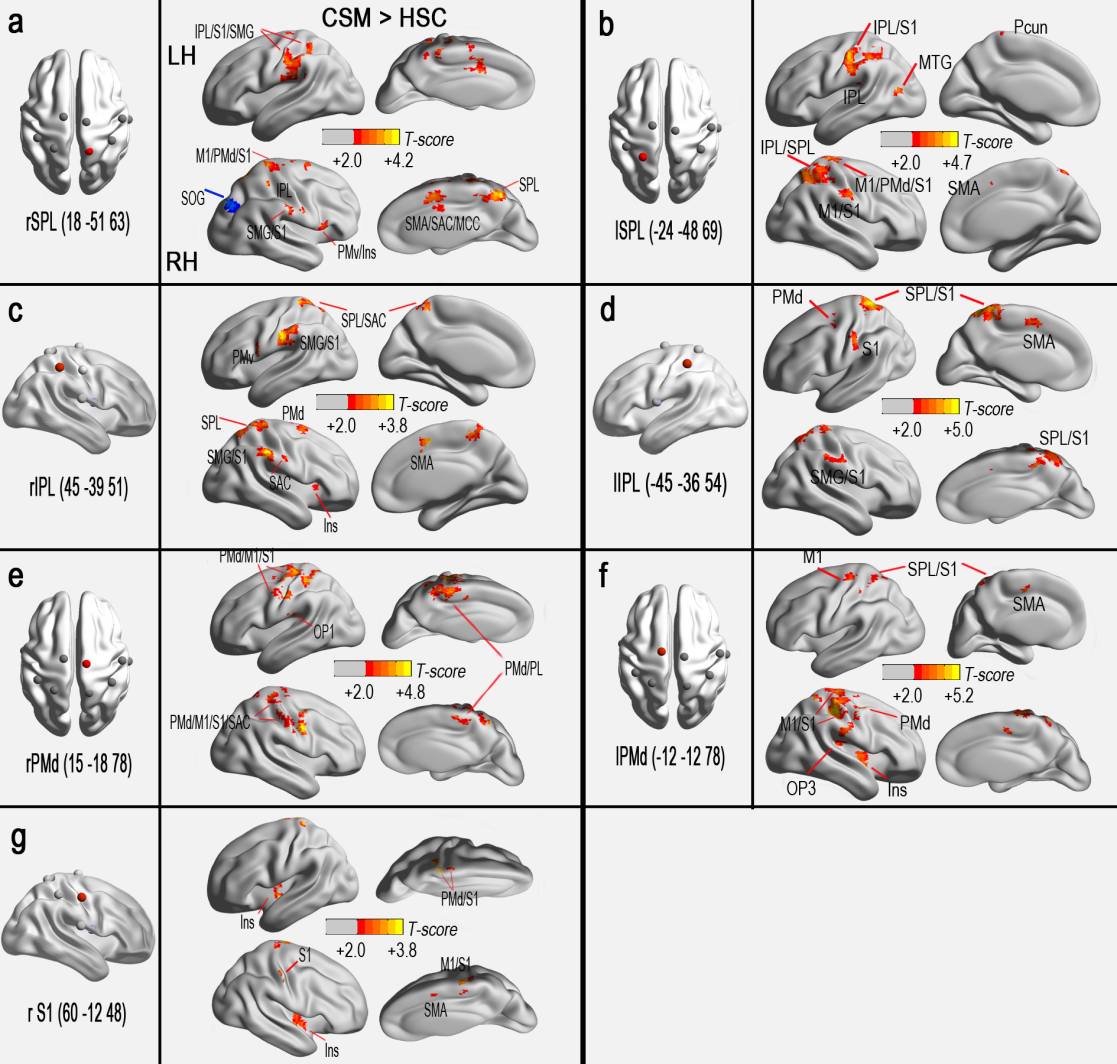
Significant group differences in the rsFC patterns of the seed regions with increased FCS (CSM > HSC, P < 0.05. The AlphaSim corrected critical cluster size was k = 23 in Fig 5a, k = 23 in Fig 5b, k = 21 in Fig 5c, k = 21 in Fig 5d, k = 21 in Fig 5e, k = 21 in Fig 5f, k = 22 in Fig 5g). The colour-bar indicates the t-scores. Yellow-red colours denote increased rsFC in patients.

The results of one-sample t-tests revealed that the rsFC network patterns of all seed regions showed significant group differences in the FCS between the CSM and HSC groups (FDR corrected; *P* < 0.001) (Figures 4 and S2). In a voxel-based GLM analysis, compared with the HSC group, the CSM group demonstrated significantly reduced rsFCs in seed regions (left PMv/PrCO and right OP4) from areas with decreased FCS (Figure 4 and Table S1, AlphaSim corrected, *P* < 0.05). The CSM group also displayed significantly enhanced rsFCs in seed regions (left or right IPL, left or right PMd, left or right SPL, and right S1) from areas with increased FCS (Figure 5 and Table S2, AlphaSim corrected, *P* < 0.05), except for a decreased rsFC cluster, the right superior occipital gyrus (SOG), that connected to the right SPL (Figure 5c).

Specifically, the FA values at the C2 level were negatively correlated with the correlation coefficient (CC) values between the left PMv/PrCO and left PMd (*r* = −0.416, *P* = 0.020), between the right IPL and right PMd (*r* = −0.360, *P* = 0.043), and between the right SPL and right premotor ventral/insula (PMv/Ins; *r* = −0.402, *P* = 0.025); however, the FA values at the C2 level were positively correlated with the increased CC values between the right S1 (BA3b) and right S1 (BA2/1) (*r* = −0.413, *P* = 0.021). In addition, the reduced JOA scores were associated with a connectivity coefficient between the right S1 (BA3b) and right Ins (*r* = −0.365, *P* = 0.042).

## Discussion

To the best of our knowledge, this study is the first to use FCS mapping to investigate functional connectivity changes in the sensory-motor network of CSM patients at rest. This study revealed the following regarding functional plasticity within the SMN: (1) decreased FCS in the operculum-integrated regions (right OP4 and left PMv/PrCO), in which the CSM group showed significantly decreased rsFC regions around the Rolandic sulcus; (2) increased FCS in the premotor (bilateral PMd), right S1, and parietal-integrated regions (bilateral IPL and SPL), in which the CSM group mainly showed significantly increased rsFC; (3) worsening JOA scores associated with decreased FCS in the operculum-integrated regions (right OP4 and left PMv/PrCO) and right S1; (4) decreased FA values at the C2 level that were negatively correlated with rsFC between the premotor regions and integrated regions but positively correlated with the rsFC inter-region of the somatosensory network (right S1_BA3b and S1_BA2/1). These myelopathy-related findings indicated that reorganisation in the strength and density of rsFC, reflecting remarkable brain plasticity, was closely associated with cervical cord injury and clinical presentation.

In the CSM group, decreased FCS was observed in the right OP4 and left PMv/PrCO, and this was positively correlated with the JOA score. However, the OP4, the ventral portion of the somatomotor strip, was more closely integrated with the areas responsible for basic sensorimotor processing and action control, and with other types of modal integration^19^,^20^. This study revealed a weaker processing ability in the OP4 with a reduced connection with the left PMd or right MTG, as shown by seed-based rsFC analysis. The former (PMd) was located in the premotor processing pathway^19^, and the latter (MTG) was associated with the retrieval of explicit information concerning actions in the alternate condition^21^. Previous anatomical and functional connectivity studies have revealed that the OP4 incorporates sensory feedback into motor actions^19^,^22^, particularly in movement-related tactile object recognition and manipulation^23^. Similar connectivity reductions in the SMN have been identified in sensory-deprived patients, and they have been suggested to represent a possible reason for the functional loss of sensory-guided movements^24^. The decreased connectedness of the OP4 in this study may help us understand the defects in movement precision, the decreased finger dexterity, and the loss of motor skills in CSM.

The PMv/PrCO is the junction region of the premotor and operculum areas, and it appears to play a key role in the integrative process. The PMv/PrCO receives input via the OP4 and is involved in polymodal action recognition, locating one's own limb in the peripersonal space, subjective limb ownership and improving action execution and understanding^25^,^26^,^27^. In this study, decreased FCS of the left PMv/PrCO also exhibited reduced rsFC in the ipsilateral OP4/OP3, S1 (representation of the arm/hand), PMd, and SMA, contralateral PMd/SMA. Decreased activity of the PMv/PrCO has been detected in a previous study of “rubber hand illusion^27^”, and a similar perception loss also exists in myelopathy patients. Together with the right OP4, the reduced connectedness of the left PMv/PrCO identified in this study may be the anatomical basis for these deficits. Incorporating additional detailed sensory-motor behavioural data is recommended to further investigate the alterations that underlie certain intrinsic neuronal activities.

We also noted that the CSM group showed significantly enhanced FCS in the right S1, bilateral PMd, and parietal-integrated regions (bilateral IPLs and SPLs). Particularly, the right S1 exhibited increased rsFC with the adjacent region (S1, BA2/1), the right M1/S1, the left PMd/S1, the bilateral SMA and the bilateral Ins. The S1 at area BA3b, covering the “hand region” in this study, is one of the major regions affected in CSM patients^28^. BA3b receives dense somatosensory inputs from the nucleus pulposus (NP) of the thalamus and relays these inputs to BA1 (texture information) and BA2 (size and shape)^29^. One explanation for the loss of afferent sensory conditions may be the less efficient funnelling of neural processing under reduced sensory impulse conduction, which might reflect an adaptive mechanism. Another explanation is that functional reorganisation may be a mere negative “side effect” that is followed by structural injury to inhibitory interneurons. Our results in the S1 support the adaptive plasticity with less efficiency hypothesis based on the following observations: (1) a more significant increase in FCS at the S1 was observed in myelopathy patients with a high JOA score compared to those with a low JOA score; (2) high levels of rsFC and directional information flow were observed in the intra-region of the S1 (directional effective connectivity is shown in the Supplementary information and Figure S3); (3) cortical evidence (grey matter volume loss) from spinal cord injury^30^. In addition, recent studies have shown widespread spatial integration in the S1 based on feedback connections from higher-level regions^31^. Surprisingly, the brain could require functional rearrangements with fascinating cross-modal plasticity when coping with sensory loss^32^.

In addition, the bilateral PMds, IPLs, and SPLs also exhibited extensively increased rsFC with the M1, sensory, and supplementary motor regions and with other premotor and integrated regions (Figure 5a-f), with the exception of reduced rsFC between the right SPL and right SOG (Figure 5c). The PMd receives its major inputs from the dorsolateral, prefrontal and superior parietal lobules, and the PMd plays a role in guiding reaching or movement sequencing^33^. Enhanced activation is observed in the PMd, where compensatory changes have been demonstrated in patients with brain injury^34^ and spinal cord injury^35^. In fact, Dong et al.^5^ also reported enhanced motor-related activation in CSM patients with a loss of dexterous finger movements as a compensatory cerebral adaptation. In this study, the IPL and SPL are both parietal-integrated regions. The IPL is involved with spatial perception and the interpretation of sensory information^5^, while the SPL is involved with spatial orientation and the receipt of sensory input from the hands^36^. Task-related enhancement in the SPL or IPL of myelopathy patients may accompany the postoperative recovery of function after cervical cord injury^5^,^7^. Interestingly, in this study, decreased rsFC was also found between the right SPL and right SOG, a fact that could influence spatial-related motion processing in CSM patients because this pathway is involved with stereomotion processing^37^.

In this study, we supposed that functional reorganisation or plasticity in the SMN could alleviate the symptoms of an impaired cervical cord but that this effect is finite to compensate for (limited) neurological dysfunction. As mentioned previously, increased FCS in the right S1 correlated with the JOA score, and a similar relationship was observed between reduced JOA scores and enhanced rsFC among the right S1 (BA3b) and right Ins. We should note that the insula is an important processing area for tactile information^38^,^39^, and increased functional connectivity in the insula and S1 (resulting from alterations in plasticity) could contribute to the discrimination of complex tactile stimuli. Similar findings in patients with selective deafferentation (sensory ganglionopathy) support these opinions^39^.

Sensory-motor cortical plasticity, which is the dynamic potential of the brain to reorganise following secondary injury in the progression of spinal cord injury^35^, has also been explored in previous studies of myelopathy patients^4^,^5^,^6^. In this study, we observed that the FA values at the C2 level were negatively correlated with increased rsFC in the intra-somatosensory regions (S1, BA3a, and BA2/1). However, these values were positively correlated with increased rsFC in the motor-related regions (i.e., the right SPL and right PMv/Ins, the right IPL and right PMd, the left PMv and left PMd). One potential explanation for this effect is that more activity coherence in the motor-related regions may be required to compensate for the decreased motor nerve conduction in the cervical cord that is observed in myelopathy. Conversely, the rsFC of sensory regions was observed to be positively correlated with damage to the cervical cord, most likely because of incomplete deafferentation due to myelopathy, a condition that can reduce the intensity and precision of sensory information impulses. Regarding CSM, chronically decreasing or blurred tactile and other somatosensory stimuli (texture, size and shape) input to the cortex results in sensory uncertainty. In this context, patients also exhibit a decreased ability to perform motor decision marking and motor spatial planning due to impaired feedback control of sensory-guided movements^1^,^3^,^32^. In this process, increased synchronisation in specific structures is used to alleviate this deficit, which appears to be finite in this study.

In addition, we reported a trend or moderate relationship between the FA values at the C2 level and the mean-normalized FCS in the left IPL (negative correlation) or the right S1 (positive correlation). These findings also support the idea that intrinsic functional plasticity results from micro-structural damage to the cervical cord.

In contrast, the lack of a significant correlation between the FCS or rsFC values and other clinical metrics, such as the NDI score, suggests that evaluating functional changes using the NDI score may be dominated by local insults to the cervical cord, not plasticity in the cortex. We also did not observe a significant correlation between the FA values at the most severe level and the FCS or rsFC values. The plausible mechanism of cerebral structural damage in CSM patients was more similar to the degeneration observed at the C2 level than it was to the degeneration at the most severe level^40^. The FA values at the C2 level reflected the fact that CSM-associated demyelination and axonal damage spread from a more caudal area of stenosis in chronic pathological processing^40^,^41^.

Several technical and biological limitations in the present study must be acknowledged. First, the increased FCS of the S1 only occurred on the right side. Asymmetrical spinal cord compression or asymmetrical spinothalamic sensory loss may explain this phenomenon. Second, we did not observe significantly altered FCS in the M1. Increased activation in the M1 provides a measurement of brain responses to controlled stimuli in task-related fMRI, such as recruitment to compensate for decreased motor nerve conduction in the spinal cord^5^,^6^, rather than intrinsic functional connectedness in SMN. Another interpretation, that heterogeneous injury in the cervical cord affects the cortex in M1, could explain the non-significant difference in M1. Due to the relatively small sample size, this study was not classified by either single- or multi-level compression or by upper or lower extremity impairments; thus, further studies with larger samples and/or additional classifications should be conducted. Finally, this study was focused on alterations in the SMN. The SMN mask was obtained from healthy controls using the Group ICA Toolbox (http://mialab.mrn.org/data/index.html). Although other SMN masks may identify different FCS levels in undiscovered regions, using a different mask should not change the overall results reported in this study. FCS calculations were independent of image mask except for the voxels near the mask boundary.

In summary, the results presented herein provide a relatively complete picture of the functional reorganisation through which insufficient sensory and motor function in CSM patients may widely affect sensorimotor functional activities. In CSM, we observed not only decreased FCS in the operculum-integrated regions (PMv/PrCO, OP4) but also increased FCS in the premotor and parietal-integrated regions, along with the right S1. Worsening JOA scores were associated with decreased FCS in the operculum-integrated regions and were also associated with connectedness in the right S1. Furthermore, the alterations of rsFC in the SMN were associated with decreased FA values at the C2 level. This knowledge may improve our understanding of the comprehensive functional defects found in patients with CSM in addition to task-related cortical activation.

## Methods

This study was approved by the Institutional Review Board of the First Affiliated Hospital, Nanchang University, China. This study was performed in accordance with approved guidelines, and it was conducted in compliance with the principles of the Declaration of Helsinki. All of the subjects provided written informed consent before beginning any study procedure.

### Subjects

Patients with cervical spondylotic myelopathy (CSM) were recruited from the First Affiliated Hospital of Nanchang University between May 2013 and June 2014, and well-matched healthy subject controls (HSCs) from the local community participated in this study. The following inclusion criteria were used for patients in this study: (1) voluntarily enrolling in the study; (2) right-handed; (3) clear evidence of cord compression on cervical spine MRI, such as cervical spondylosis, herniated discs or ligamentum flavum hypertrophy, and (4) demyelination with hyper-intensity of the cord on T_2_-weighted images (T_2_WI). Two radiologists determined spinal cord compression when the cord surface was clearly indented or the cord diameter was narrowed by compression. The following exclusion criteria were employed: (1) refusal by the patient to enrol; (2) trauma or infection related to cord compression; and (3) other spinal or brain neurological disorders, such as multiple sclerosis, or a history of neurological disorders.

The clinical severity of myelopathy was evaluated using the JOA score system^42^ and NDI questionnaires. The JOA score system evaluates the severity of myelopathy by assigning scores based on the degree of dysfunction, and the NDI was designed to measure the activities of daily living in patients with neck pain. The participants were excluded if the maximum displacement in one or more of the orthogonal directions (x, y, z) was > 2 mm or if the maximum rotation (x, y, z) was > 2.0° in the maximum head motion of the rs-fMRI data. Finally, 31 patients and 31 healthy subjects were included in the present study. Detailed demographic and clinical data of all of the subjects are shown in Table 1.

### Image acquisition

A SIEMENS Trio 3.0 Tesla MRI scanner was used to acquire the MR images. (1) Brain fMRI in the resting state requires all subjects to remain as still as possible, to not think systematically, to not fall asleep, and to keep their eyes closed. An echo planar imaging (EPI) sequence was used with the following parameters: repetition time/echo time (TR/TE) = 2000/30 ms; field of view (FOV) = 200 × 200 mm; matrix = 64 × 64; slice thickness = 4 mm; slice gap = 1.2 mm; duration = 8 min and 6 s. (2) Axial DTI images covering the cervical spinal cord from C1 to C7 were acquired to evaluate cervical cord structural damage^43^, and the spin echo single-shot echo planar sequence parameters were as follows: TR/TE = 5000/106 ms; FOV = 128 × 124 mm; NEX = 2; matrix = 128 × 124; slice thickness = 5 mm; 20 nonlinear diffusion-weighted gradient directions with *b* = 600 s/mm^2^ and one additional image without diffusion weighting (i.e., b = 0 s/mm^2^). (3) Sagittal and axial conventional T_1_-weighted, T_2_-weighted and T_2_-FLAIR (fluid-attenuated inversion recovery) images were acquired in the brain and cervical spinal cord for the diagnosis in each subject.

### Functional data preprocessing

fMRI data preprocessing was conducted using the Data Processing Assistant for Resting-State fMRI Advanced Edition (DPARSFA) V2.3 (http://www.restfmri.net) in the MATLAB platform (The MathWorks, Inc., Natick, MA, USA). The initial images from each subject were discarded to eliminate magnetic saturation effects, and the remaining 230 images were slice-time corrected, motion corrected, spatially normalised to the standard Montreal Neurological Institute (MNI) EPI template and resampled to 3×3×3 mm^3^ cubic voxels. Finally, the normalised images were smoothed with full-width-half-maximum (FWHM) Gaussian kernel (6 mm), and temporal band-pass filtering (0.01–0.08 Hz) was applied to reduce low-frequency drift and physiological high-frequency noise. Nuisance linear regression was performed using white matter, CSF, and six head motion parameters as covariates.

### Functional connectivity strength analysis

In the present study, the functional connectivity strength (FCS) was measured with weighted network degree centrality by mapping the degree of functional connectivity across the sensory-motor network (in Eq (1), N _voxels _ = 14,106)^11^. 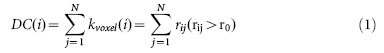

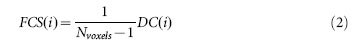
where the *r_ij_* is the correlation coefficient between voxel *i* and voxel *j* and *r_0_* represents the correlation threshold value that is set to eliminate weak correlations^11^,^17^,^18^. Different *r_0_* values (*r_0_* = 0.1, 0.15, 0.2, 0.25, 0.3, 0.35, 0.4) were considered in this study, and the *k (i)* of each voxel was divided by the individual global mean of *k_0_* within the SMN-mask. A functional template from the “Medical Image Analysis (MIA) Lab (http://mialab.mrn.org/data/index.html)”, was used to normalise and reduce the effect of individual variability. The individual data were then converted using Fisher's Z-transformation for group comparisons and analysed using the “REST-DC” program in the REST V1.8 package (http://www.restfmri.net). Notably, the FCS metric is referred to as the ‘‘degree centrality'' of weighted networks in terms of graph theory and usually indicates its central role in the functional integrity of the networks (for review, see^11^). The SMN primarily included the following brain areas: the bilateral precentral and postcentral gyrus, the bilateral supplementary motor area and inferior frontal gyrus, a portion of the bilateral middle cingulate cortex and paracentral lobule, a portion of the bilateral inferior parietal lobule and supramarginal gyrus, and part of the bilateral insula.

### Fractional anisotropy (FA) metrics calculation in the cervical spinal cord

FA metrics were calculated in the DTI native space for each subject using the Diffusion Toolkit, which is one component of the TrackVis (http://www.trackvis.org/) software package. Regions of interest (ROIs) were typically placed at the C2 vertebra level and the level of the most severe cervical canal stenosis.

### Statistical analysis for group differences in FCS

The FCS map comparison was performed using a general linear model (GLM) and standard statistical parametric mapping (SPM8, http://www.fil.ion.ucl.ac.uk/spm), one way analysis of covariance (ANCOVA) with age and gender as covariates followed by post-hoc two-sample t-tests with the SMN mask. The significance level was set at a corrected *P* value of 0.05, and multiple comparisons were corrected by Monte Carlo simulations using the AlphaSim program with the REST package and the following parameters: for an individual voxel, *P* = 0.01, FWHM of smoothing = 6 mm, rmm = 5, and iterations = 1000.

### Statistical analysis for the connectivity strength-clinical relationship

Linear regression (in SPSS) was performed to assess the relationship between the clinical metrics (JOA, NDI or FA values in the cervical cord) and FCS of the obtained regions with significant group differences, controlling for gender and age. All statistical analyses were performed using SPSS, and the statistical significance level was set at *P* < 0.05 and corrected for multiple comparisons using the Bonferroni correction.

### The rsFC networks linking with the influenced FCS regions by CSM and statistics

To reveal the specific networks that were influenced by CSM, the peak coordinates of the regions with altered FCS were selected as the coordinates of the seed's centre based on the results of the FCS analysis. The mean time course of a sphere with an 8-mm diameter centred at the peak coordinate of each seed region was extracted in both the CSM and the HSC groups. *Pearson's* correlation coefficient (CC) between the mean time series of each seed region and that of each voxel in the entire brain was computed and converted to *z*-values to improve normality. Next, the individual *z*-values were entered into a random-effect one-sample *t*-test in SPM8 to identify brain regions that exhibited significant positive correlations with each seed region for each group (voxel-wise *P* < 0.001; cluster-wise FDR corrected *P* < 0.001), as rsFC networks in CSM and HSC groups. Finally, a mask was generated by combining the regions of rsFC networks in the CSM and HSC groups for group comparisons .

The rsFC networks comparison was performed using a GLM and the standard SPM8 toolkit, one way analysis of covariance (ANCOVA) with age and gender as covariates followed by post-hoc two-sample t-tests with the mask of combining network. The significance level was set at a corrected *P* value of 0.05 (AlphaSim correction running in the REST package and the following parameters: for an individual voxel, *P* = 0.01, FWHM of smoothing = 6 mm, rmm = 5, and iterations = 1000) to identify regions of altered connectivity in the patients with CSM.

Finally, a linear regression analysis was performed in CSM patients to assess the relationship between the clinical metric and average rsFC measured in the abnormal rsFC region between the groups (*P* < 0.05, Bonferroni corrected).

## Author Contributions

**Author contributions statement** F.Q.Z. and H.H.G. formulated, conceived and designed the research. L.W. and Y.M.T. collected MRI data samples. F.Q.Z. and Y.Z. preprocessed and analysed the data. F.Q.Z. and L.C.H. contributed reagents/materials/analysis tools. F.Q.Z. and Y.Z. wrote the manuscript. All authors reviewed the manuscript.

## Supplementary Material

Supplementary Information Supplementary Information

## Figures and Tables

**Figure 1:**
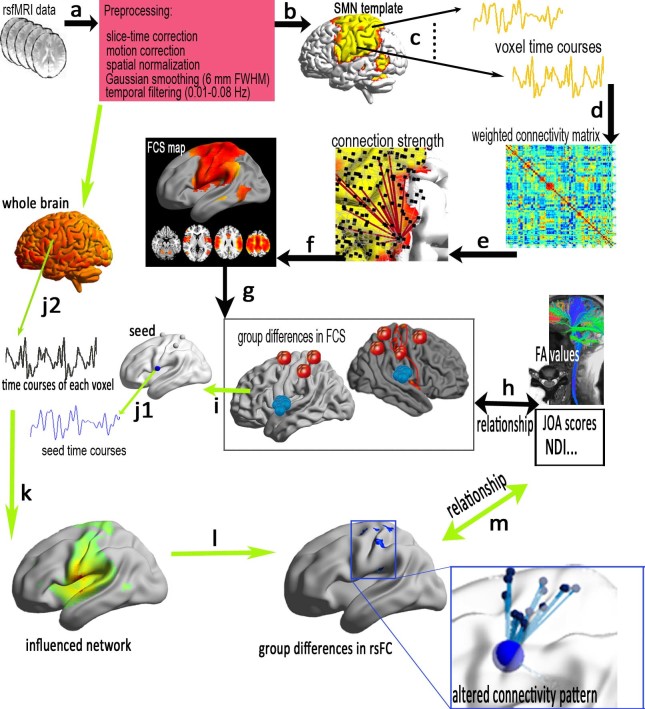
A flowchart of voxel-based intrinsic functional connectivity strength (FCS) and the influenced connectivity pattern in the sensory-motor network (SMN). (a) Preprocessing of subject's resting-state fMRI data; (b) using a SMN mask for FCS analysis; (c)the time series of each voxel in the SMN template was extracted in MNI-space, and (d) then *Pearson's* correlation was used to construct the voxel-based weighted connectivity matrix; (e) for calculations of the connection strength, different *r_0_* values (*r_0_* = 0.1, 0.15, 0.2, 0.25, 0.3, 0.35, 0.4) were considered in this study; (f) the FCS map was constructed within the SMN; (g) group comparison of the FCS and(h) clinical metrics associated with the altered FCS of the SMN. To reveal the specific networks that were influenced by CSM, (i) the peak coordinates of regions with altered FCS were selected as the coordinates of the seed's centre, then time series was extracted in a spherical ROI of the seed (8-mm diameter, j1) and each voxel of the whole brain (j2); (k) calculated functional connectivity correlations and constructed influenced network, and a group comparison between the CSM and HSC groups (l); finally, statistical analysis of the relationship between the clinical metric and the average rsFC measured in the abnormal rsFC region between the groups (m).

**Table 1:** Demographic data and clinical measure scores for the cervical spondyloticmyelopathy group and healthy controls.

Subject		CSM	HSC	*P*-value
n		31	31	n/a
Age		51.38 ± 6.33	50.91 ± 6.31	0.764
Gender (male/female)		22/9	22/9	1.0
Handedness (right/left)		31/0	31/0	n/a
Duration of symptoms (month)		6.84 ± 7.85 (1–36)	n/a	n/a
NDI scores		0.316 ± 0.114	0.011 ± 0.001	< 0.0001
JOA scores		11.45 ± 2.45	17 ± 0	< 0.0001
Motor upper	</p>	2.19 ± 0.70	4 ± 0	< 0.0001
Motor lower	</p>	2.80 ± 1.30	4 ± 0	< 0.0001
Sensory deficit	</p>	3.48 ± 0.96	6 ± 0	< 0.0001
Bladder dysfunction	</p>	2.97 ± 0.18	3 ± 0	< 0.0001
FA values				
FA values at the C2 level	</p>	0.613 ± 0.050	0.673 ± 0.052	0.005
FA values at the most severe level	</p>	0.511 ± 0.078	0.662 ± 0.045*	0.002

Note: BA = Brodmann area; C = cervical vertebra; CSM = cervical spondylotic myelopathy; FA = fractional anisotropy; HSC, healthy subject control; IPL = inferior parietal lobule; JOA = Japanese Orthopaedic Association; L = left; MNI = Montreal Neurological Institute; n/a = not applicable; NDI = Neck Disability Index; OP4 = operculum parietale 4; PMv = premotor ventral; PrCO = precentral operculum; PMd = premotor dorsal; R = right; S1 = primary somatosensory complex; SPL = superior parietal lobule.* mean FA values of whole cervical cord. The same abbreviations are used for all tables.

**Table 2:** Significant differences in the functional connectivity strengths between the CSM and HSC groups (CSM *vs.* HSC)

				MNI coordinates			
Brain regions		BA	Peak T-scores	X	y	z	Cluster size (voxels)
CSM < HSC							
PMv/PrCO	L	6/44	−3.58	−54	−9	6	99
OP4	R	42/43	−2.89	63	−15	12	55
CSM > HSC							
IPL	L	40	3.15	−45	−36	54	87
	R	40	2.67	45	−39	51	39
SPL	L	5/7	2.77	−24	−48	69	52
	R	5	2.92	18	−51	63	23
PMd, caudal	L	6	3.77	−12	−12	78	70
	R	6	2.72	15	−18	78	26
S1 (BA3b, hand)	R	3	2.92	60	−12	48	20
