# Supplementary material for: Intrinsic Functional Plasticity of the Sensory-Motor Network in Patients with Cervical Spondylotic Myelopathy
Source: Sci Rep. 2015 Apr 21;5:9975. doi: 10.1038/srep09975 (PMC4404678; doi:10.1038/srep09975)
Supplement: Supplementary Information [file srep09975-s1.pdf]

Intrinsic Functional Plasticity of the Sensory-Motor Network in Patients with Cervical Spondylotic Myelopathy

F.Q. Zhou [1*,2,4], Y.M. Tan[1], L. Wu[1,2], Y. Zhuang[3], L.C. He[1,2], H.H. Gong [1*,2]

[1]Department of Radiology, the First Affiliated Hospital, Nanchang University, Nanchang, Jiangxi Province, 330006, PRC, [2]Jiangxi Province Medical Imaging Research Institute, Nanchang, Jiangxi Province, 330006, PRC, [3]Department of Oncology, The Second Hospital of Nanchang, Nanchang, Jiangxi Province, 330003, PRC, [4]Department of Orthopaedics and Traumatology, Li Ka Shing Faculty of Medicine, The University of Hong Kong, Pokfulam, Hong Kong

Supplementary information:

Effective connectivity revealed by Granger causality analysis (GCA) in the abnormal rsFC regions

To improve our understanding of the relationship between the clinical symptoms and directional information flow in the abnormal rsFC regions within sensory-motor network (SMN) in patients with Cervical Spondylotic Myelopathy (CSM), we hypothesize that altered effective connectivity patterns among the abnormal rsFC regions would be observed in patients with CSM. In this study, Granger causality analysis (GCA) was used to analyse the effective connectivity and to understand the directional information flow between the abnormal rsFC regions due to connectivity rearrangements or new contacts in CSM patients.

Methods

Effective connectivity was analysed by Granger causality using REST-GCA in the REST toolbox[1]. Significant differences in FCS between the two groups were defined as x (seed regions). The abnormal rsFC regions linking with the influenced FCS regions by CSM were defined as y. Effective connectivity was calculated based on the direct influence of x on y ($F_{x \rightarrow y}$) and of y on x ($F_{y \rightarrow x}$). Next, the residual-based F was normalised (F') and standardised to the Z score for each voxel ($Z_{x \rightarrow y}$ and $Z_{y \rightarrow x}$, subtracting the global mean F' values, divided by the standard deviation)[1]. Finally, the information flow of abnormal rsFC was investigated between the two groups using two-sample t-tests in SPSS 13.0.

Results

Compared to the HSC group, the CSM group showed increased directional effective connectivity from the left PMv/PrCO to the left PMd (t = 2.878, P = 0.006) or left S1 (t = 2.120, P = 0.038), from the left SMG/S1 to the left IPL (t = 2.056, P = 0.044), from the right S1 (BA3b) to the right S1 (BA1/2) (t = 2.186, P = 0.033), from the right SPL to the right IPL (t = 2.048, P = 0.045), and from the right IPL to the right SPL (t = 2.043, P = 0.045). Compared to the HSC group, the CSM group also showed increased bidirectional effective connectivity between the left IPL and left S1 (t = 2.201, P = 0.032; t = 2.234, P = 0.029, respective), between the right IPL and right PMd (t = 3.272, P = 0.002; t = 2.364, P = 0.021, respectively), and between the right IPL and bilateral SMA (t = 3.022, P = 0.004; t = 2.261, P = 0.027, respectively). Comparatively, patients with CSM also showed a significant decreas in effective connectivity from the right SPL to the right SOG (t = -3.602, P = 0.001) (Figure S3).

Discussion

The Granger causality analysis revealed altered effective connectivity patterns among the regions of altered connectedness in the patients with CSM. The enhanced rsFC regions affected by CSM showed an increased directional effective connectivity to the left ventral integrated regions (PMv/PrCO) or bidirectional effective connectivity between the premotor/supplementary motor and parietal integrated regions (IPL). This increased information flow showed somatosensory integration and backward circuitry as reorganisation to improve the precision of movement. We also observed

decreased effective connectivity from the right SPL to the right SOG companion with reduced rsFC. Reduced information concerning spatial orientation and sensory input from the hand was suggested to appreciably impact stereomotion processing.

The S1 acted as afferent receptors. Under the condition of low sensory stimulation, neuronal increased intrinsic activity in the S1 could enhance the efficiency as an adaptive mechanism. A neural-level explanation of our study may be that the S1 (including the right SMG/S1) exhibited enhanced hemodynamic responses and causal interactions with dorsal integrated regions. In addition, the causal flow observed from the right S1 (BA3b) to the S1 (BA1/2) was consistent with the classical understanding of information flow within the S1[2]. CSM-related effective alteration in both the sensory and motor systems revealed that the information flow referred to myelopathy in the cervical cord and replenishment of intrinsic neuronal activity.

1 Zang, Z. X., Yan, C. G., Dong, Z. Y., Huang, J. & Zang, Y. F. Granger causality analysis implementation on MATLAB: a graphic user interface toolkit for fMRI data processing. *J Neurosci Methods* **203**, 418-426 (2012).

2 Wang, Z. et al. The relationship of anatomical and functional connectivity to resting-state connectivity in primate somatosensory cortex. *Neuron* **78**, 1116-1126 (2013).

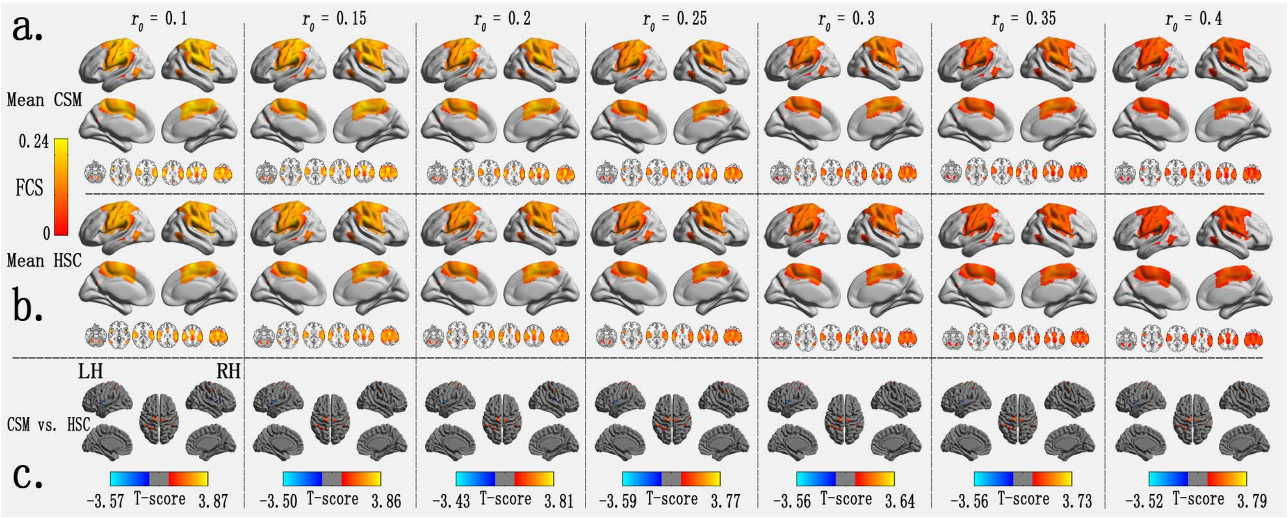

Figure S1 Within- and between-group FCS maps derived at different correlation thresholds (r_0).

(a-b) Mean FCS maps within CSM and HSC groups using different correlation thresholds (r_0 = 0.1, 0.15, 0.2, 0.25, 0.3, 0.35, 0.4). (c) Z-statistical difference maps between the 2 groups. Notably, a similar pattern was observed in most of the regions that showed CSM-related FCS's changes derived at different thresholds.

Note: CSM = cervical spondylotic myelopathy; FCS = functional connectivity strength; LH = left hemisphere; RH = right hemisphere; HSC = healthy subject control; r_0 = correlation threshold values. The same abbreviations were used for all of the figures and tables.

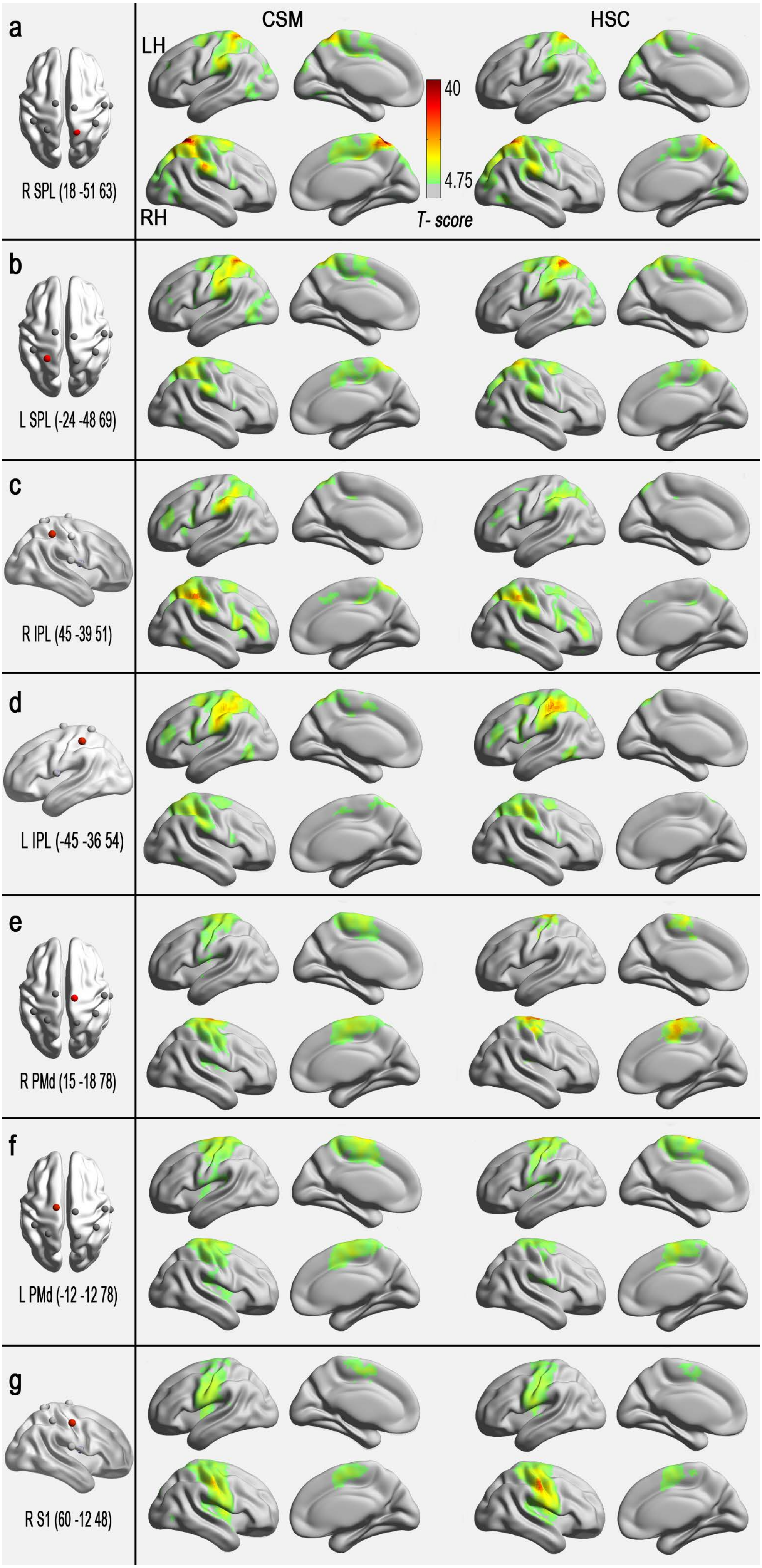

a
b
c
d
e
f
g
CSM
HSC
LH
RH
40
4.75
T- score
R SPL (18 -51 63)
L SPL (-24 -48 69)
R IPL (45 -39 51)
L IPL (-45 -36 54)
R PMd (15 -18 78)
L PMd (-12 -12 78)
R S1 (60 -12 48)

Figure S2 Significantly positive rsFC patterns in the seed regions of increased FCS in both the CSM and HSC groups ($P < 0.001$, FDR corrected).

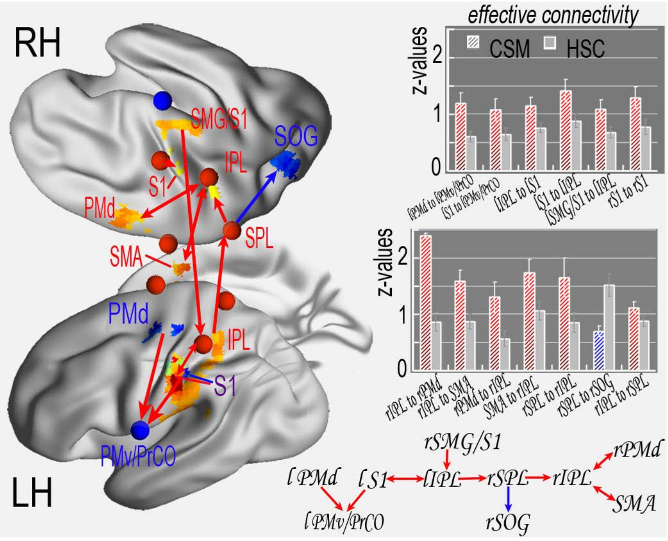

Figure S3 Altered effective connectivity among abnormal rsFC regions in the sensory-motor network of myelopathy patients. The left and lower right columns show the schematic overview of the changes in effective connectivity, which was measured using the Granger causality, in CSM patients compared with healthy controls. The red arrows indicate increased effective connectivity, and the blue arrows indicate decreased effective connectivity in the CSM patients. The upper right column shows the mean z-values of effective connectivity and the standard errors.

Table S1 Significant rsFC differences in seed regions of decreased FCS (CSM > HSC)

Brain regions	BA	Peak T-scores	MNI coordinates			Cluster size (voxels)
			x	y	z	
Seed from left PMv/PrCO						
left OP4/OP3	43	-3.91	-48	-18	18	52
left S1(arm)	2	-3.10	-54	-24	48	74
left S1(hand)	3	-2.89	-30	-36	63	40
left PMd	6	-3.31	-33	-6	60	32
left SMA	6	-2.76	-12	-12	66	31
R PMd/SMA	6	-3.32	21	0	66	59
Seed from right OP4						
left PMd	6	-2.91	-36	-18	33	56
right MTG	21	-3.81	60	0	-9	31

Note: CSM = cervical spondylotic myelopathy; FCS = functional connectivity strength; IPL = inferior parietal lobule; k/k0 = normalised FCS; MTG = middle temporal gyrus; MNI = Montreal Neurological Institute; OP3 = operculum parietale 3; OP4 = operculum parietale 4; Pcun = precuneus; PL = paracentral lobule; PMv = premotor ventral; PrCO = precentral operculum; PMd = premotor dorsal; S1 = primary somatosensory complex; SAC = somatosensory association cortex; SMA = supplementary motor area; SMG = supramarginal gyrus; SOG = superior occipital gyrus; SPL = superior parietal lobule. The same abbreviations were used for all of the figures and tables.

Table S2 Significant rsFC pattern differences in each seed region of increased FCS between the CSM and the HSC groups (CSM > HSC)

Brain regions		BA	Peak T-scores	MNI coordinates			Cluster size (voxels)
				x	y	z	
Seed from right SPL							
SMA/SAC/MCC	R	6,7,32	4.40	12	-63	63	586
M1/PMd/S1	R	6,4,3	4.44	18	-36	78	149
SMG/S1	R	40,1	3.95	69	-21	30	133
PMv/Ins	R	44	2.94	54	15	12	52
IPL	R	40	3.04	42	-42	60	27
IPL/S1/SMG	L	40,3	4.78	-42	-45	63	424
SOG	R	19	-3.56	27	-84	45	102
Seed from left SPL							
IPL/S1	L	40,3	4.70	-48	-39	51	440
Pcun	L	7	4.15	-3	-45	78	38
MTG	L	37	3.37	-42	-72	15	30
IPL	L	41	3.15	-42	-36	27	36
IPL/SPL	R	40,7	3.75	36	-54	54	334
M1/PMd/S1	R	6,4,3	3.70	15	-33	75	111
S1/M1	R	4,3,2	3.65	63	-12	36	94
SMA	B	6	2.78	0	6	48	35
Seed from right IPL							
SMG/S1	L	2,40	4.20	-60	-21	30	234
SPL/SAC	L	7,5	3.74	-21	-48	72	164
PMv	L	44	2.84	-57	6	18	36
SAC	R	5	4.09	3	-36	60	180
SMG/IPL	R	40	4.25	60	-33	39	172
SMA	B	6	3.74	0	0	51	131
SPL	R	7	3.16	24	-63	57	41
PMd	R	6	4.08	39	60	18	33
Ins	R	13,47	2.49	36	21	3	22
Seed from left IPL							
SPL/S1	L	7,5,2	4.98	-21	-51	69	357
	R	7,2	3.57	18	-51	66	308
SMG/S1	R	40,2	3.84	69	-12	27	110
S1 (arm)	L	2	3.31	-51	-24	42	63

PMd	L	6	2.73	-30	-9	42	40
SMA	L	6	3.60	-3	0	54	67
Seed from right PMd							
PMd/M1/S1/SAC	R	6,4,3,7	4.22	54	-3	39	327
PMd/PL	B	6	3.51	0	-27	69	210
PMd/M1/S1/SAC	L	6,4,5,3	3.86	-33	-27	69	270
PMd/M1/S1	L	4,6,3	3.40	-51	-9	54	91
OP1	L	40	3.36	-60	-24	18	53
Seed from left PMd							
M1/S1	R	2,3,4	5.17	48	-30	48	684
PMd	R	6	4.69	42	-3	57	73
Ins	R		4.06	36	0	-6	46
OP3	R	13	3.49	39	-33	24	41
SMA	L	6	3.52	-6	-12	48	76
SPL/S1	L	7	3.05	-18	-54	51	70
M1	L	4	3.57	-33	-27	69	57
Seed from right S1							
PMd/S1	L	6,3	3.79	-12	-36	78	110
Ins	L	13	3.53	-39	-6	-6	31
	R	13	3.42	45	-6	-3	39
M1/S1	R	4,3	3.12	9	-24	75	74
S1	R	2,1	3.65	54	-30	57	55
SMA	B	6	3.30	0	6	57	34
	R	6	2.54	15	-18	54	26

Note: Ins = insula; IFG = inferior frontal gyrus; L = left; MFG = middle frontal gyrus; R = right; RO = rolandic operculum; rsFC = resting state functional connectivity; PreG = precentral gyrus; PostG = postcentral gyrus.
